# An objective absence data sampling method for landslide susceptibility mapping

**DOI:** 10.1038/s41598-023-28991-5

**Published:** 2023-01-31

**Authors:** Yasin Wahid Rabby, Yingkui Li, Haileab Hilafu

**Affiliations:** 1 Department of Engineering, Wake Forest University, Winston-Salem, NC USA; 2 Department of Geography & Sustainability, University of Tennessee, Knoxville, USA; 3 Department of Business Analytics and Statistics, University of Tennessee, Knoxville, USA

**Keywords:** Environmental sciences, Natural hazards, Statistics

## Abstract

The accuracy and quality of the landslide susceptibility map depend on the available landslide locations and the sampling strategy for absence data (non-landslide locations). In this study, we propose an objective method to determine the critical value for sampling absence data based on Mahalanobis distances (MD). We demonstrate this method on landslide susceptibility mapping of three subdistricts (Upazilas) of the Rangamati district, Bangladesh, and compare the results with the landslide susceptibility map produced based on the slope-based absence data sampling method. Using the 15 landslide causal factors, including slope, aspect, and plan curvature, we first determine the critical value of 23.69 based on the Chi-square distribution with 14 degrees of freedom. This critical value was then used to determine the sampling space for 261 random absence data. In comparison, we chose another set of the absence data based on a slope threshold of < 3°. The landslide susceptibility maps were then generated using the random forest model. The Receiver Operating Characteristic (ROC) curves and the Kappa index were used for accuracy assessment, while the Seed Cell Area Index (SCAI) was used for consistency assessment. The landslide susceptibility map produced using our proposed method has relatively high model fitting (0.87), prediction (0.85), and Kappa values (0.77). Even though the landslide susceptibility map produced by the slope-based sampling also has relatively high accuracy, the SCAI values suggest lower consistency. Furthermore, slope-based sampling is highly subjective; therefore, we recommend using MD -based absence data sampling for landslide susceptibility mapping.

## Introduction

Landslides are the movement of rock, soil, and earth along a slope^1^ when the shear stress on the slope materials exceeds the shear strength^2^. It causes damage to infrastructure and the loss of human lives worldwide^3–5^. Landslide inventory and susceptibility mapping are critical to mitigate the losses caused by landslides^2,6–9^. Landslide inventory documents previously occurred landslides^10^, while landslide susceptibility describes the probability of landslides over an area^11^. Landslides are affected by various causal factors, such as slope, curvature, land use/land cover, geology, and elevation^7,12,13^. Landslide inventory and its relationship with different causal factors can be used to derive the landslide susceptibility map^14^.

Various statistical methods have been used for landslide susceptibility mapping, including logistic regression, support vector machines, random forest, and gradient boosting^15–17^. These statistical methods use landslide causal factors as independent variables and landslide locations (presence data) and non-landslide locations (absence data) as dependent variables^4^. The presence data are mainly from the landslide inventory. In contrast, the absence of data are usually unavailable and requires a specific strategy to sample locations where the probability of landslide is low^7,18^. The quality and accuracy of the landslide susceptibility maps depend not only on the quality of causal factors and presence data but also on the absence data sampling method and sometimes the accuracy depends on how this sampling is conducted^18^.

Random sampling is the most common approach for the absence data. It considers all locations other than the recorded landslides for absence data^19,20^. This method requires a representative landslide inventory of the entire area^21^. It is suitable for landslide susceptibility mapping in a relatively small area but faces challenges at a large area or regional scale^12^. The accuracy of the landslide susceptibility map based on random sampling is generally low and biased toward the known landslide locations^21^. Various absence-data sampling methods have been proposed to improve the accuracy and quality of landslide susceptibility mapping, including prior data exploratory analysis, buffer-controlled sampling, distance and density-based measures like Kernel density estimation, Euclidean distance, one class or presence-only classification method, and species density distribution modeling like Bioclim^7,8,12,21^.

Prior data exploratory analysis determines a safe zone for absence-data sampling based on the available landslide locations^7,8,22^. This method generally chooses one of the most important causal factors, such as slope and geology, to determine the safe zone for the absence-data sampling^8,12^. However, the results generated using this method are biased towards the selected factor. For instance, if the safe zone is determined based on slope, the model will likely be biased towards the slope^8^. Yao et al.^23^ used a buffer-controlled sampling method, assuming that the areas near each other are more similar than those distant apart. The selection of the buffer distance is subjective because it depends on expert knowledge^21^. Hong et al.^24^ proposed a one-class classification or presence only method similar to the one-class support vector machine method. In this method, classification like absence and presence data are not given in the model's training stage. Only the presence data is used to classify an area into two parts: one part is similar to the presence data or landslides, and the other has dissimilarities with the landslides. The area with high dissimilarities is used for absence-data sampling.

Distance-based sampling assumes that areas with similar environmental conditions (explained by the causal factors) experience similar geomorphic processes like landslides^8,21^. A distance threshold, known as the critical value, is needed to determine the sampling space for absence data^19^. Although several distance-based measures have been used, determining this critical value has yet to be explained^21^. Generally, users select the critical value subjectively to maximize the accuracy of the landslide susceptibility map^8^. Moreover, only one method, like the area under the curve or Continuous Boyce Index, is used to assess the mapping accuracy^17,21^ without consideration of the mapping consistency^17,25^. A landslide susceptibility model can achieve high accuracy by increasing the area under high and very high landslide-prone zones. However, it may overestimate the landslide susceptibility by assigning landslide-free areas as prone zones^26^. Implementing the overestimated map for practical purposes is impossible as it loses its consistency^17^. Zhu et al.^21^ found that decreasing the sampling space of the absence-data increases the accuracy of the landslide susceptibility map but may overestimate the landslide susceptibility^8,21^. Choosing the critical value or threshold is essential to satisfy both accuracy and consistency.

Various sampling methods have been proposed, and each has some shortcomings. Prior data exploratory analysis can be biased method. As for the distance-based method, the selection of distance threshold has an impact on the accuracy of the landslide susceptibility map. Moreover, for slope and distance-based method various thresholds can be applied and based on the accuracy a threshold is selected, which reduces the objectivity of these methods. In this regard, there is a need for a objective method which is applicable for any part of the world and also not dependent on the variables or landslide causal factors of susceptibility mapping. To fill up this gap, in this work, we proposed an objective method to determine the critical value of absence-data sampling based on the Chi-square distribution of the Mahalanobis distance and a user-specified confidence level. We applied this proposed method to the landslide susceptibility mapping in the three Upazilas (sub-district) of the Rangamati district, Bangladesh, and compared the model performance with a traditionally used slope-based method for absence-data sampling.

## Methodology

This study employed the third law of geography^21^ to determine sampling space for absence-data sampling. According to the third law of geography, if two areas have the same geographical environment, they will experience the same geographical processes such as landslides^21^. The characteristics of the geographic environment used in this study are the landslide causal factors. Since we are searching for sampling space for (landslide) absence-data sampling, we must find out areas with the least similarities to the landslide locations. We assume that landslide locations will have a geomorphic environment defined by landslide causal factors. For example, the slope is a landslide causal factor, and for all the landslide locations, there will be a typical value of slope (e.g., the average slope for the observed landslide locations). We seek locations whose slope possesses the highest dissimilarities with the typical slope of the landslide locations. If we have *n* number of landslide locations and *p* number of causal factors, then these locations will have a mean environmental condition based on the *p* causal factors. Non-landslide locations will be farther away from that mean condition. This study employs Mahalanobis distance to measure the distance between the mean landslide condition and the condition of a potential site to determine the extent of its dissimilarity with the landslide locations.

### Mahalanobis Distance

Mahalanobis Distance (MD) is a distance metric that measures the distance between a data point location and the distribution of datasets^27,28^. MD is an extension of the Euclidean Distance metric and can improve clustering and classification algorithms^19^. The Euclidean distance measures the distance between two points in *p*-dimensional space. It works well when the dimensional spaces are independent of each other^28^. MD is a generalization of the Euclidean distance that allows for potential interdependency among the dimensional spaces by dividing the Euclidean distance with the covariance matrix^19^. More specifically, the MD of a potential point represented by a vector of causal factors *X* from the centroid representation of a landslide point cloud with mean vector *m* and a covariance matrix *C* is:

1 $$MD=\sqrt{{\left(X-m\right)}^{T }{C}^{-1}}\left(X-m\right)$$

As illustrated in Eq. (1), MD reduces the correlation of variables by dividing the distance matrix by the covariance matrix^27^. MD has been generally used in outlier detection and multi-class classifications^28^. In landslide susceptibility mapping, MD can be used to define the sampling space for absence-data. The recorded landslide locations only cover a very small portion of the study area. Therefore, a large part of the area is not classified as landslides or non-landslides^28^. Based on landslide locations and distribution of the causal factors, MD defines the similarity of an area to landslides' conditions. If the similarity is high, the area has a high chance for landslide and is not suitable for absence-data sampling.

It is, however, hard to determine if the similarity of an area is different enough for the absence-data sampling. Some studies used the 5th quantile value to define the absence sampling space^19^. Zhu et al.^21^ tested a set of user-defined thresholds to determine the appropriate value for landslide susceptibility mapping. Their work demonstrated that reducing absence sampling space continuously increases accuracy but overestimates the landslide susceptibility. However, this simple try-out strategy does not provide a statistical means to determine the optimal threshold value for absence-data sampling.

We proposed an approach to offer a statistical means for determining the MD threshold for absence-data sampling. The MD is a normalized quantity. If the causal factors have a distribution that the *p*-variate Gaussian distribution can approximate, the MD follows a Chi-squared distribution with *p-1* degrees of freedom. Furthermore, even if the causal factors do not have an approximate *p*-variate Gaussian distribution, the MD has an approximate Chi-squared distribution with *p−1* degrees of freedom, as long as the number of causal factors is large enough (Nader et al.). Based on this assumption, a critical value can be determined for a specified significance level, such as the commonly adopted significance level of 0.05. For example, if we use 15 causal factors in our study, the critical value of the MD, i.e., an MD beyond which we would conclude a potential non-landslide location is a viable sample, is 23.69. That is, when the MD is greater than this critical value, it is considered as an outlier or different enough from the rest of the data^27^. Therefore, we use such a critical value to determine the locations for absence-data sampling.

**Figure 1:**
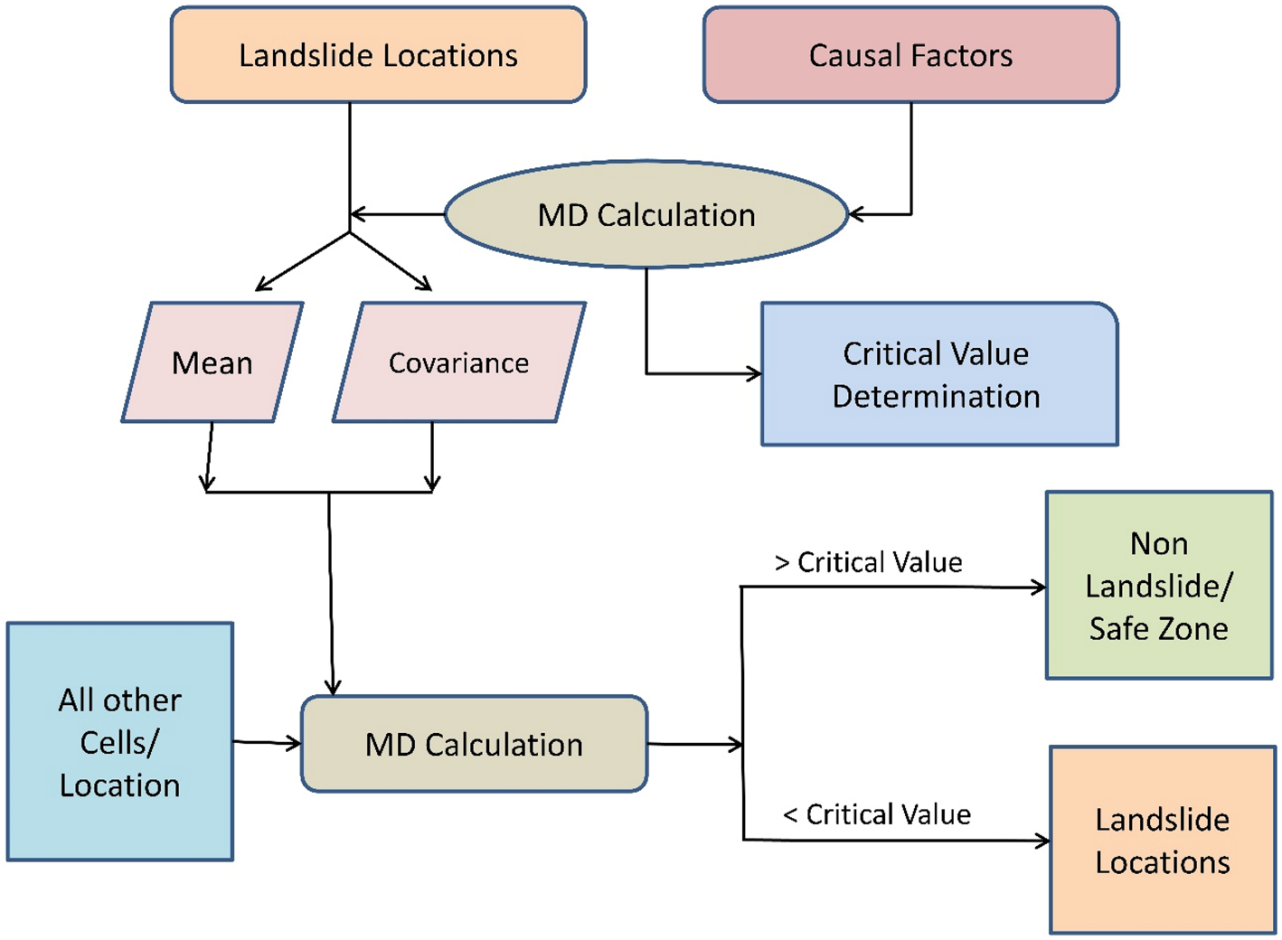
Flow chart of the MD based absence-data sampling.

Figure 1 shows the flow chart of our proposed method. As stated above, *n* represents the number of available landslide locations, and *p* represents the number of causal factors. A critical value is determined based on the *p−1* degrees of freedom. This critical value determines if a new point or location is a potential candidate for absence-data sampling. For any new candidate location, MD was calculated based on the mean value and the covariance matrix of the distribution of the causal factors of the *n* landslide locations. A location or point with an MD value greater than the critical value is designated as a safe zone for absence-data sampling.

To demonstrate the efficiency of this proposed method, we applied it to the landslide susceptibility mapping on three Upazilas of the Rangamati district, Bangladesh, and compare its derived landslide susceptibility map with the map produced based on a traditional slope-based method for absence-data sampling.

## Case study

### Study area and landslide inventory

**Figure 2:**
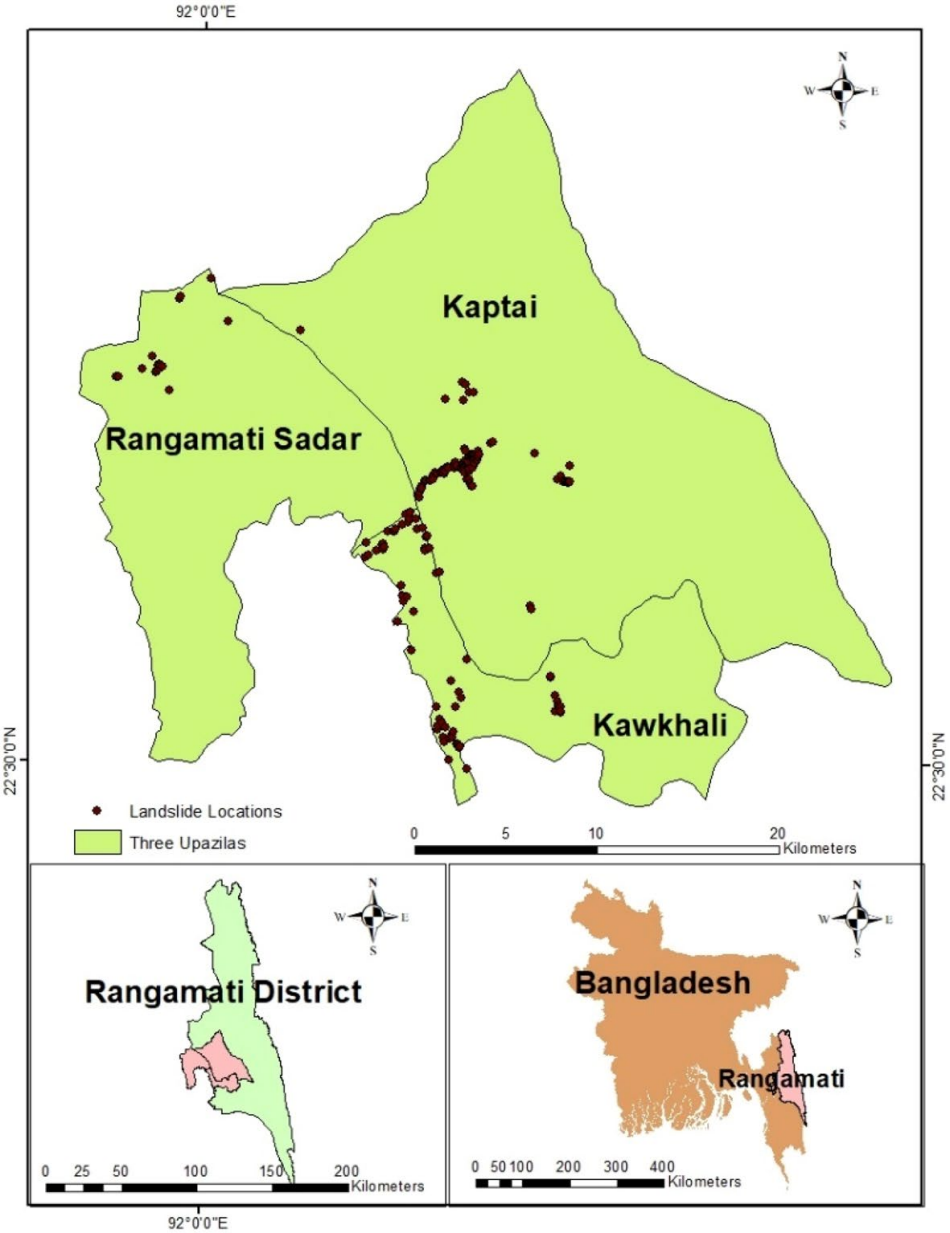
Study area: locations of three Upazilas (Rangamati Sadar Kaptai and Kawkhali).

**Figure 3:**
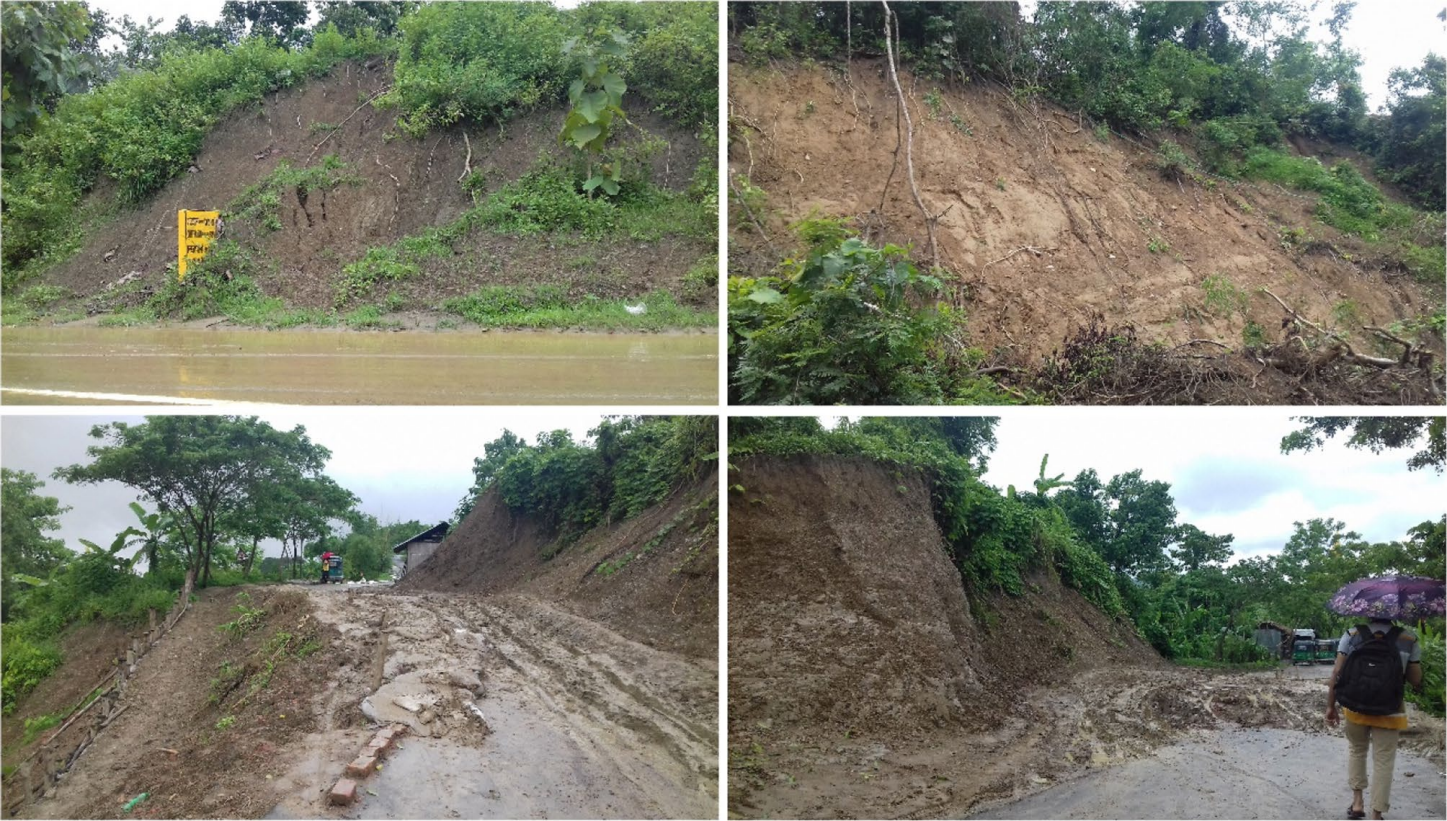
Pictures of some of the landslides in the study area (Pictures were taken by the Researchers during July 2017).

This study focused on three Upazilas of the Rangamati district, Bangladesh: Rangamati Sadar, Kaptai, and Kawkhali (Fig. 2). Rangamati Sadar is the largest city in this area. In June 2017, more than 100 people were killed by landslides (Fig. 3) in this district, and these three Upazilas were the most affected areas^29^. This district covers 1145 km^2^^30^ with an elevation range from 7 to 576 m above mean sea level and a slope range from 0° to 52°. The western part of the area has a comparatively gentle slope, while the west and central regions are relatively steep. The bedrock of this area comprises several geological formations, including Dihing, Dupitila, Girujan Clay, Bhuban, Bokabil, and Tipam Sandstone^31^. Most of the area is covered by natural vegetation or plantation agricultural fields. Plantation agriculture and unplanned land use/land cover changes create conducive conditions, and intensive rainfall triggers landslides in this area^6,25^.

A total of 261 landslide locations (Fig. 2) were recorded from January 2001 to January 2019. These landslides were collected by^32^ based on the integrated field and Google Earth mapping and Rabby et al.^31^ based on Google Earth mapping.

### Landslide causal factors

**Figure 4:**
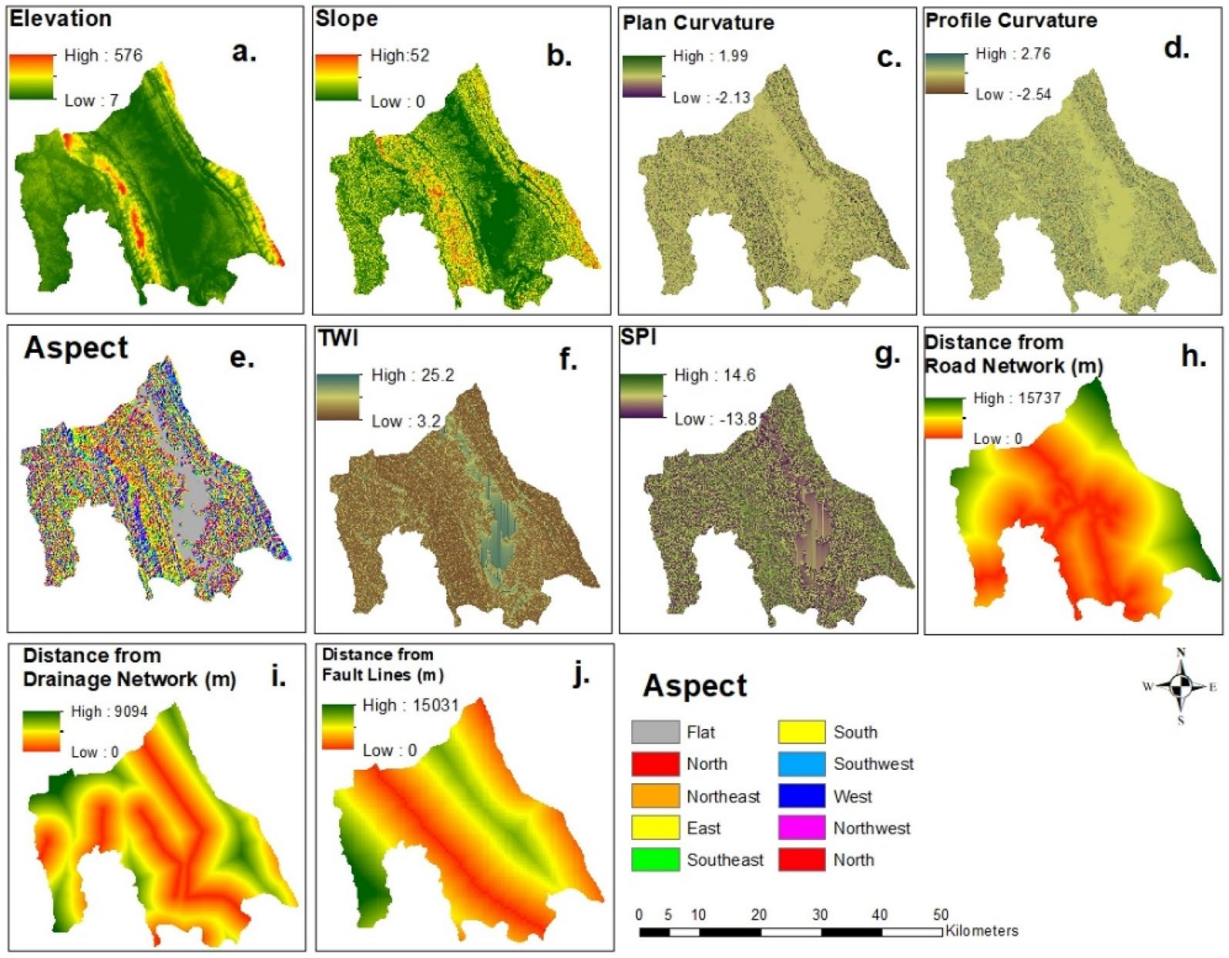
Landslide causal factors: (**a**) elevation; (**b**) slope; (**c**) plan curvature; (**d**) profile curvature; (**e**) aspect; (**f**) TWI; (**g**) SPI; (**h**) Distance from the road network; (**i**) distance from the drainage network; (**j**) distance from fault lines (modified from^25^).

**Figure 5:**
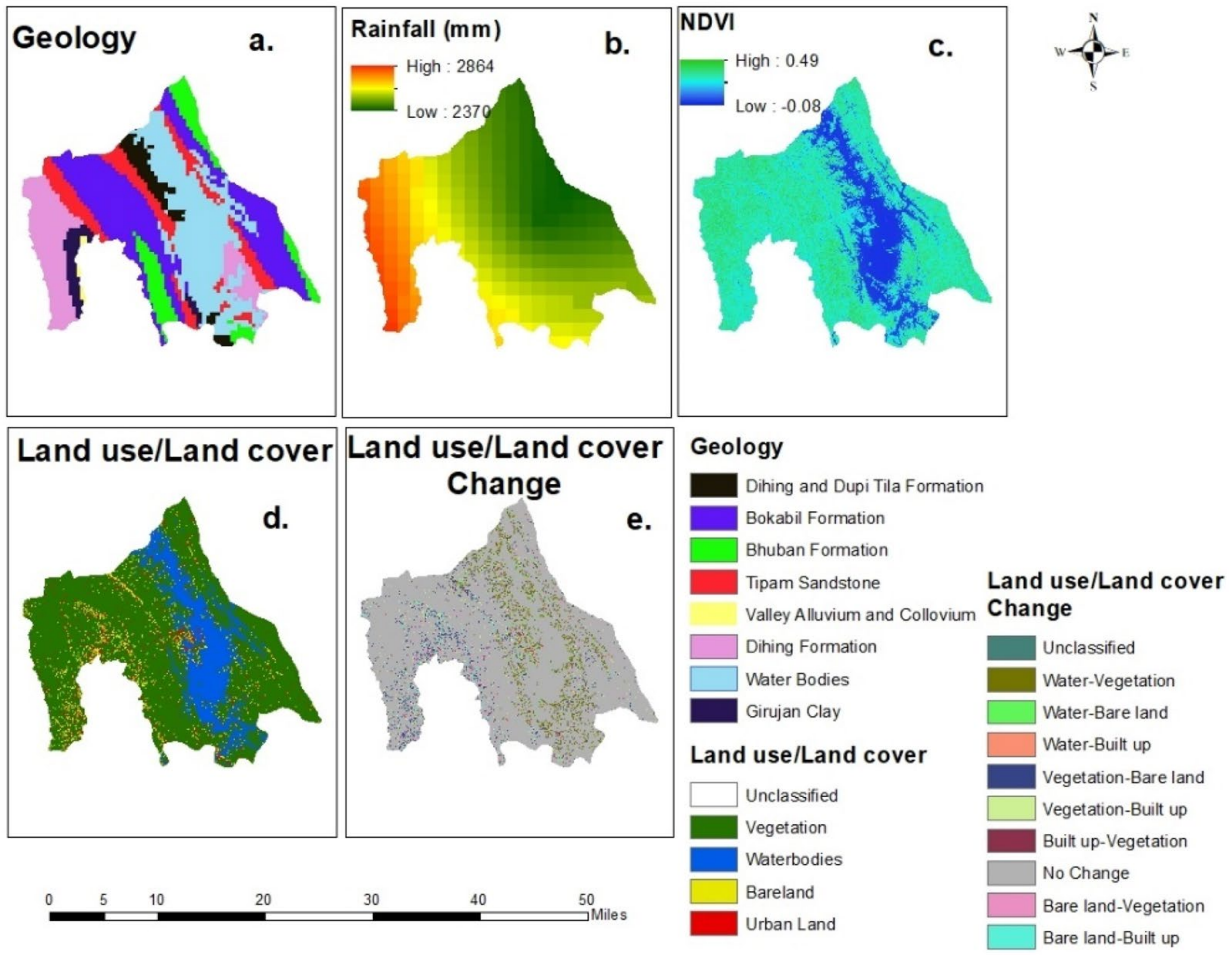
Landslide causal factors: (**a**) Geology; (**b**) Rainfall; (**c**) NDVI; (**d**) Land use/land cover; (**e**) land use/land cover change (modified from^25^).

We used 15 landslide causal factors for landslide susceptibility mapping (Figs. 4 and 5) based on the availability of data and previous literature^29,33^. The raster maps of these factors were prepared by Abedin et al.^29^, and we modified the maps using Arcmap 10.8. Table 1 lists the factors, resolutions, types, and data sources of these raster maps. Since the resolution of most factors is 30-m, we selected 30-m as the resolution for the landslide susceptibility mapping.

**Table 1:** Landslide causal factors used in this study.

Factor name	Type	Resolution	Reasons to choose
Elevation	Geophysical	30 m	Geomorphic, environmental, and anthropogenic processes depend on elevation^34^
Slope	Geophysical	30 m	With the increase of slope probability of slope failure increase^35^
Plan curvature	Geophysical	30 m	Affects the concentration of water over the surface after rainfall and thus can control the pore pressure of the soil^36^
Profile curvature	Geophysical	30 m	Affects the concentration of water over the surface after rainfall and thus can control the pore pressure of the soil^36^
Aspect	Geophysical	30 m	Aspect involves how much sunlight an area will receive. Consequently, it has effects on several geomorphic processes, including erosion and evapotranspiration^35^
TWI	Hydrological	30 m	Represents stream power of erosion^34^
SPI	Hydrological	30 m	Represents stream power of erosion^34^
Distance from Road Network	Anthropogenic	1000 m	Road construction in the hilly areas alters the structure of the landscape, increasing the probability of landslides^34^
Distance from drainage network	Hydrological	1000 m	The probability of landslide is generally high near the stream network^35^
Distance from the fault lines	Geological	1000 m	Fault lines show the zones of weakness where the probability of landslide is high^32^
Geology	Geological	1000 m	Geological formations: Dihing and Dupi Tila are susceptible to landslides^6^
Rainfall	Hydrological	1000 m	Excessive rainfall in a short time acts as a triggering factor^12^
Normalized difference vegetation index (NDVI)	Environmental	30 m	It shows the vegetation health and in a vegetated surface probability of landslide is low^34^
Land use/land cover (2018)	Environmental	30 m	One of the main driving factors of landslides in the study area^29^
Land use/land cover change	Environmental	30 m	The rate of land use land cover change is high in the study area which creates conducive condition for landslides^31^

### Absence-data sampling

**Figure 6:**
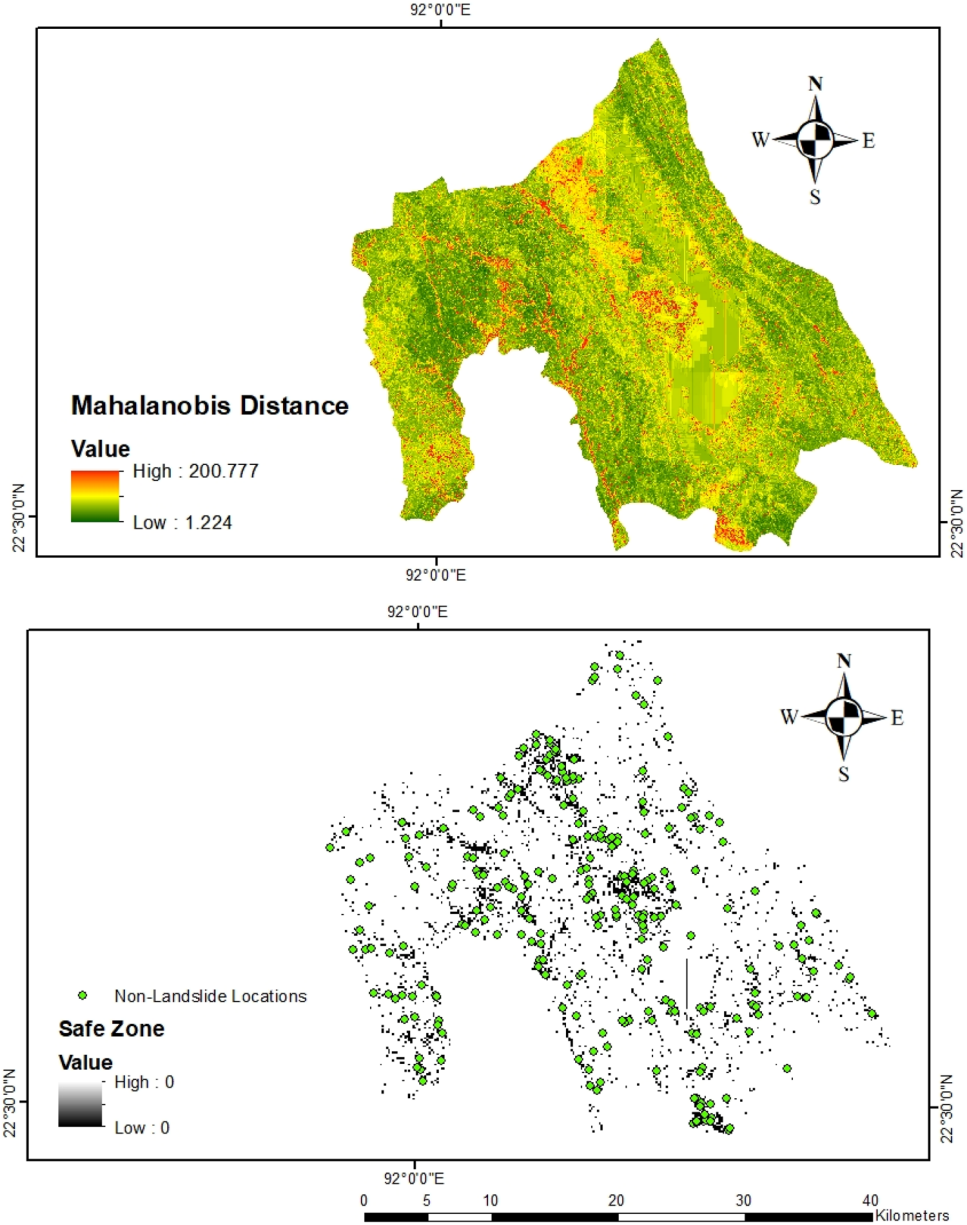
Spatial distribution of Mahalanobis distance (MD) and sampling space (Maps were produced using ArcMap 10.8).

We computed the MD values for all landslide locations based on the 15 causal factors. MD values ranged between 1.2 and 200.8 (Fig. 6). The degree of freedom for the approximate Chi-square distribution of MD values based on these 15 factors is 14, resulting in a critical value of 23.69 for the significance level of 0.05. We calculated the MD value for each location based on the mean and covariance matrix derived from the landslide locations. We then applied this critical value to determine the sampling space for the absence-data of (Fig. 6). Specifically, the locations whose MD values are greater than the threshold are used for absence-data sampling to generate 261 absence-data randomly.

**Figure 7:**
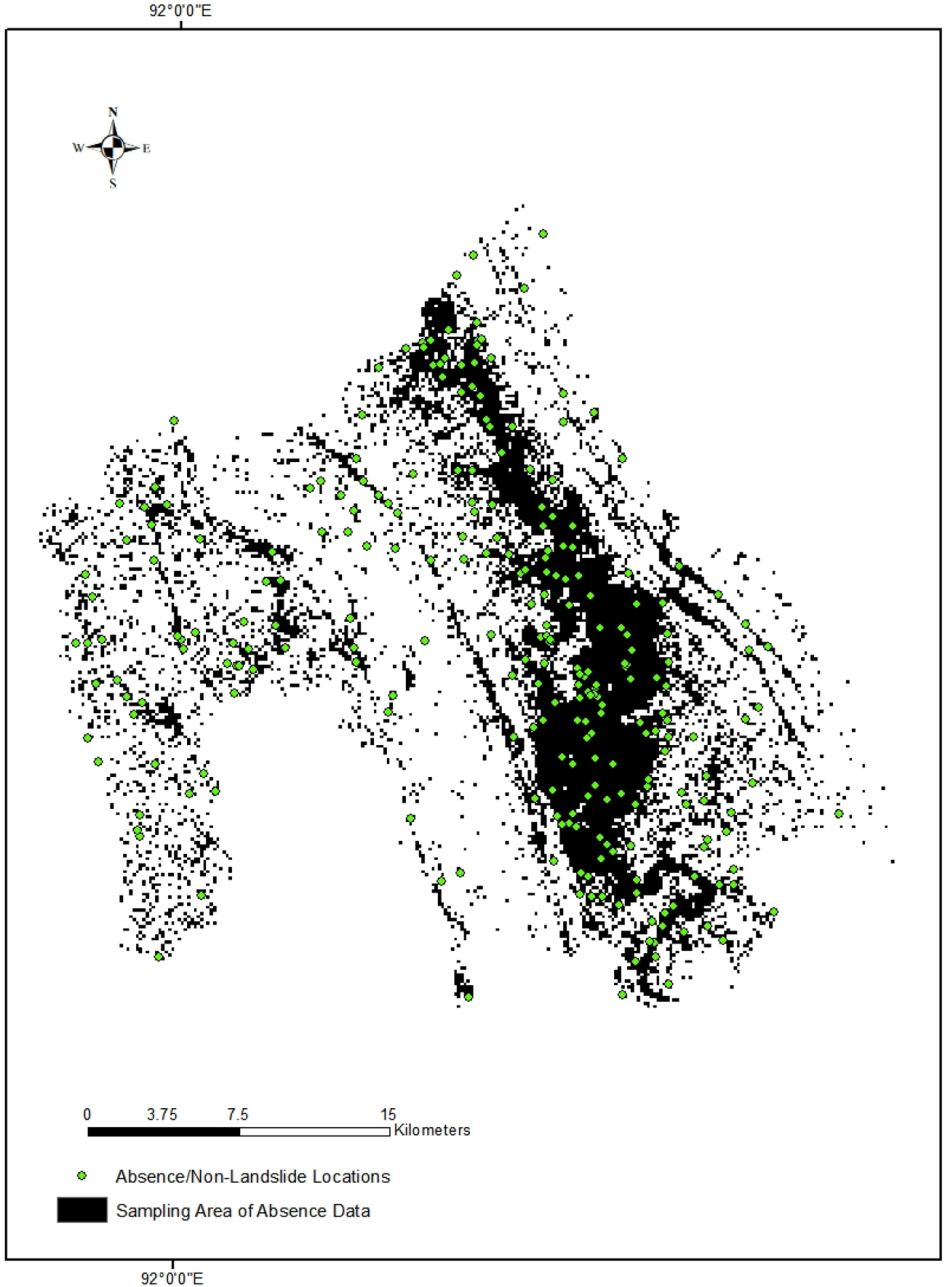
Absence-data sampling area based on different thresholds of slope (Maps were produced using ArcMap 10.8).

For comparison, we also used a slope-based sampling to determine the low landslide probability area for absence data^34^. The slope threshold is determined based on expert knowledge and judgment. Adnan et al.^29^ used the slope threshold of < 2° for absence-data sampling in the Cox’s Bazar district of Bangladesh. Ali et al.^37^ determined areas where slope < 3° for absence-data sampling in their study in the Kysuca river basin of Slovakia. We used a threshold of slope < 3° to randomly sample the 261-absence-data (Fig. 7).

### Landslide susceptibility mapping

We used the random forest model to produce the landslide susceptibility maps. The random forest model proposed by Breiman^38^ is an ensemble learning method^39^. Bootstrap aggregation is employed in RF to select subsets of observations. It generates a set of decision trees^21^ and decorrelates the trees^39^. The ensembles of decision trees decided the class membership of the dependent variables based on the highest number of votes^40^. While training the model, instead of using all the predictors, RF uses a random sample of predictors^39^. There can be a couple of strong predictors in a study, and in splitting the trees, these predictors will have an influence. RF uses a subset of predictors to overcome this problem^21^. Since all the datasets are not used in modeling, the unused data are known as out-of-bag (OOB)^40,41^. These unselected datasets are used in determining the error and importance of the predictors in the model^39^. We used the "randomForest" package in R to develop the RF model for the landslide susceptibility mapping^42^.

As described earlier, we generated the same number of non-landslide locations (261). This produced a dataset of 522 (261: presence data; 261 absence-data). We divided the dataset into training (391: 75%) and validation datasets (130:25%) for the landslide susceptibility mapping. In the MD-based sampling method, we used all 15 factors for the landslide susceptibility mapping. We did not include slope in the landslide susceptibility mapping for the slope-based method because the absence-data were sampled based on the slope threshold.

### Evaluation of the model performance and consistency

#### Performance assessment

We use statistical index-based measures: true positive rate (TPR) (sensitivity), true negative rate (TNR) (specificity), and Kappa index. TPR is the proportion of landslide locations that were classified correctly as landslide locations by the model. TNR is the proportion of absence-data that are correctly classified as absence-data by the model^7^. Kappa index (Eq. 2) is the ratio of observed and expected agreement, representing the model's reliability^7,40^.

2 $$Kappa=\frac{{P}_{obs }-{P}_{exp}}{1- {P}_{exp}}$$

where P_obs_ = observed correct classification rate, P_exp_ = expected correct classification rate

3 $${P}_{obs =} \frac{TP+TN}{n}$$

4 $${P}_{exp =} \frac{\left(TP+FN\right)(TP+FP)(FP+TN)(FN+TN)}{\sqrt{N}}$$

where TP = true positives (landslide locations classified as landslide locations by the model); TN = true negatives (non-landslide locations classified as non-landslide locations by the model); FN = false negatives (landslide locations classified as non-landslide locations by the model); FP = false positives (non-landslide locations classified as landslide locations by the model); n = proportion of pixel that are classified correctly; N = the number of total training locations; Kappa index ranges from 0 to 1 where 0 indicates the agreement occurred due to random guess, whereas 1 indicates a perfect agreement.

The statistical index-based measures above are computed using a posterior threshold value of 0.5. That is, if the estimated posterior probability of a location being a landslide location, given its observed values of causal factors, exceeds 0.5 then the model classifies it as a landslide location. Otherwise, it classifies it as a non-landslide location. However, a threshold value of 0.5 could be excessive and these metrics are not very ideal for risk profiling landslide locations. For this reason, we also use the receiver operating characteristics (ROC) curve for assessing model performance. ROC curve is a graphical representation of a models’ classification performance at different posterior probability threshold values^35^. It is produced by plotting the false positive rates (FPR) on the X-axis and the true positive rates (TPR) on the y-axis obtained from a grid of posterior probability threshold values. To compare the models, we used the area under the ROC curves (AUC), which shows the area in terms of the percentage of area under the graph^43^. The training dataset was used for assessing model fitting performance, whereas the validation dataset was used to evaluate the model prediction performance^17^. AUC values for the ROC curve range from 0 to 1. The greater the value, the better is the model in risk profiling landslide locations. Generally, AUC > 0.7 is considered as fair model, and AUC < 0.5 indicates that the model classifies the data randomly^13,44^.

#### Consistency assessment

The seed cell area index (SCAI) proposed by Suzen and Doyuran^45^ was used for the consistency assessment of the models. SCAI is the ratio between the areal extent of susceptibility classes and the percentage of landslides that occurred in the susceptibility classes and can be described as Eq. (5).

5 $$SCAI= \frac{{N}_{i}}{{n}_{i}}$$

where N_i_ = percentage of area under *i* susceptibility class; n_i_ = percentage of landslides under *i* susceptibility class.

SCAI value ranges from 0 to ∞. The smaller the SCAI value, the more consistent the model is. SCAI value decreased from low to high susceptibility zones^46^. This index determines whether landslide locations or pixels spread over a conservative areal extent^47^. It can identify if a model overestimates landslide susceptibility. An overestimated landslide susceptibility map tends to classify most areas as high susceptibility zones (the percentage of high susceptibility zones is comparatively higher than other zones).

## Results

### Variable importance of the causal factors

**Figure 8:**
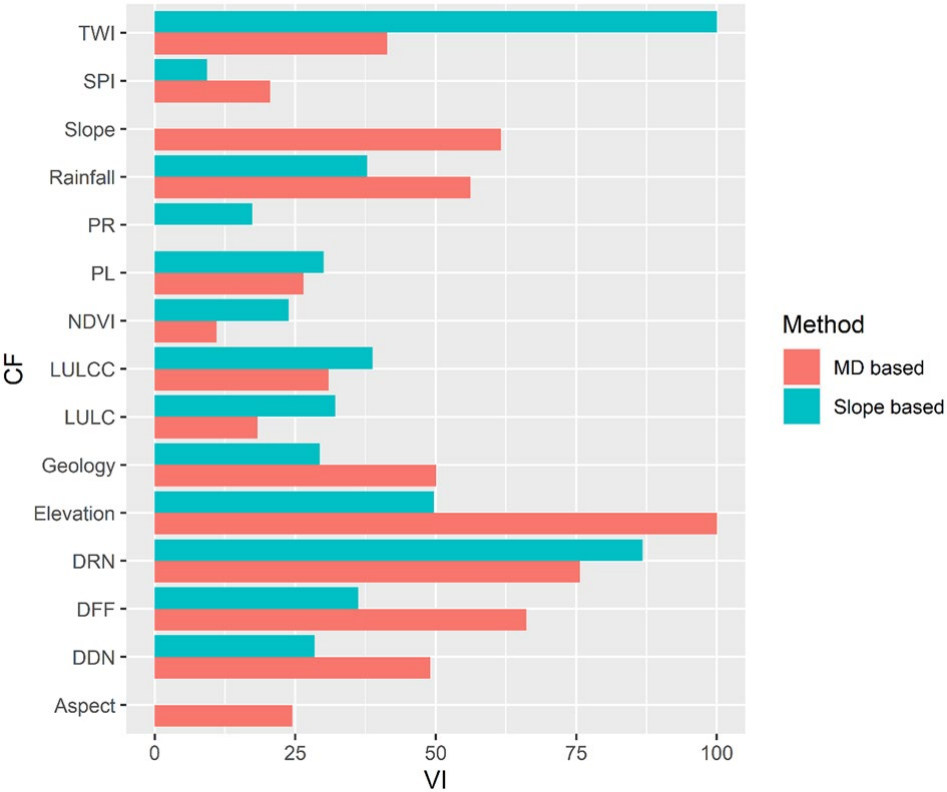
Variable importance plot of random forest model. *CF* causal factors, *VI* variable importance, *PR* profile curvature, *PL* plan curvature, *LULCC* land use/land cover change, *LULC* land use/land cover, *DRN* distance from road network, *DFF* distance from fault lines, *DDN* distance from drainage network.

Variable importance shows which causal factors have the most predictive power in a random forest model^8^. In our proposed MD-based sampling method (Fig. 8), elevation (100.0) is the most important causal factor, followed by the distance from drainage network (75.7), distance from the fault lines (66.1), slope (61.6) and geology (50.1). Factors like profile curvature (0.0), NDVI (11.0) has the least importance in the model.

In the slope-based sampling (Fig. 8), TWI (100.0) is the most important causal factor, followed by the distance from the road network (86.8) and elevation (49.7). TWI is a slope-related index. It becomes the most important causal factor because the absence-data was determined by the slope threshold and the slope factor was removed from the landslide susceptibility model. Factors like aspect (0.0), SPI (9.3), and PR (17.4) were the least critical causal factors. SPI is another slope-related index; because TWI has already become an essential causal factor, another slope-related index is likely less important in the model. The comparison of the two methods indicates that different sampling methods result in different variable significance. In MD-based sampling, elevation is the most important causal factor, while it is the third most important causal factor in the slope-based sampling method. In MD-based sampling, comparatively smaller areas were used for absence-data sampling, but the sampling space spread over the whole area. On the other hand, in the slope-based sampling, only Kaptai lake, its nearby areas, and the areas with gentle slopes in the southwest were designated for absence-data sampling. Even with the same landslide locations, the use of different absence-data sampling methods produces different landslide susceptibility maps.

### Landslide susceptibility maps

**Figure 9:**
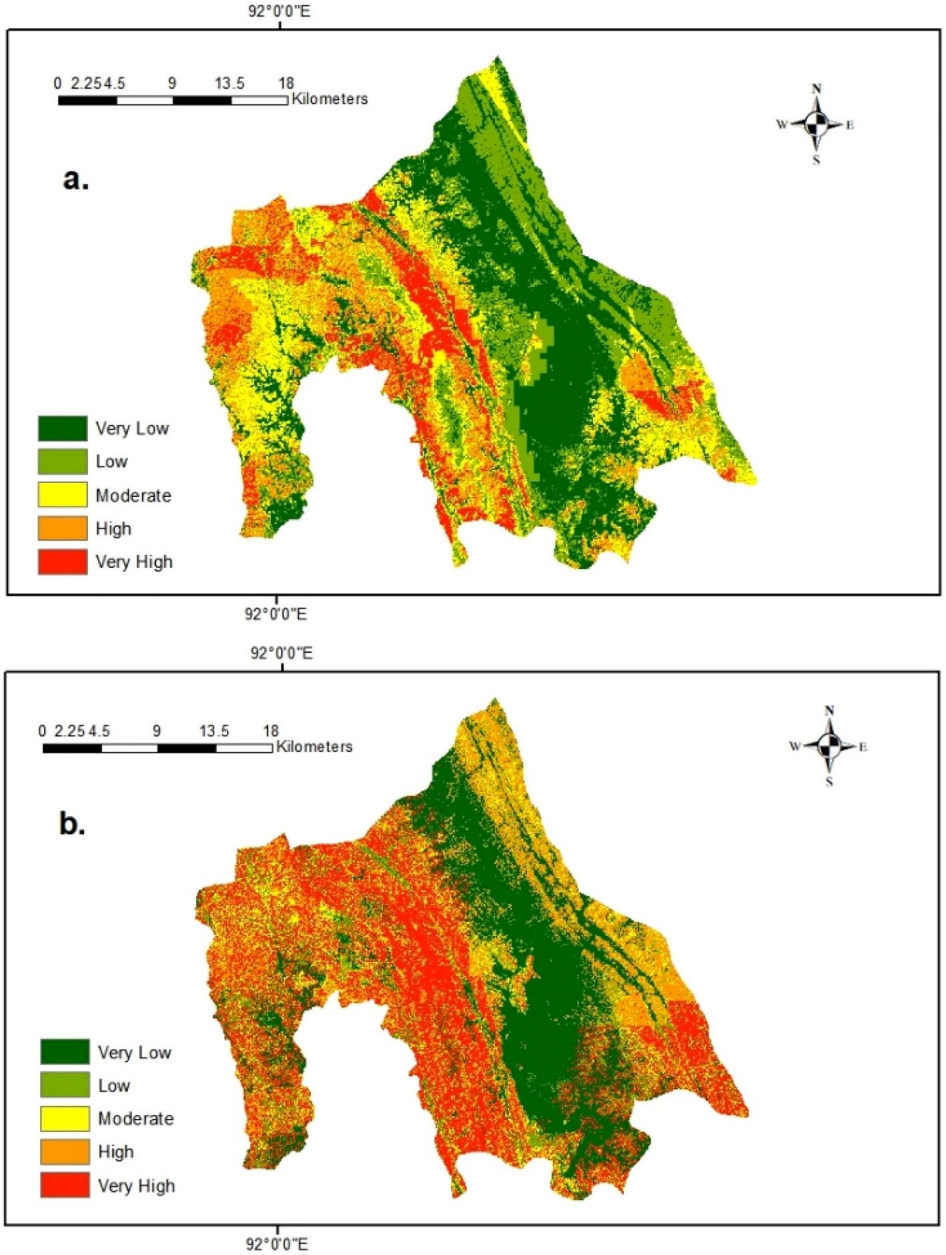
Landslide susceptibility maps based on the random forest model using: (**a**) Mahalanobis distance based absence-data sampling; (**b**) Slope-based absence-data Sampling (Maps were produced using Arcmap 10.8).

Each landslide susceptibility map provides landslide probabilities from 0.0 to 1.0. We used a natural break method to classify the landslide probabilities into five susceptibility zones (Fig. 9): very low, low, moderate, high, and very high.

In the landslide susceptibility map produced using our proposed MD-based sampling, valleys in the southeast areas (Fig. 9) near the Rangamati Lake were classified as low or very low susceptibility zones. High and very high susceptibility zones spread around the surrounding areas of the landslide locations. The high susceptibility zones in the northwest of the study area contain the Chittagong-Rangamati highway because the distance from the road network has higher variable importance in the model. Elevation and slope are the other two important causal factors. As a result, the areas on higher elevations and steeper slopes were classified as high or very high susceptibility zones. The distance from fault lines is another causal factor with high variable importance in the model. The fault lines in this area stretch from northwest to south-west; thus, the areas near those fault lines were classified as high or very high susceptibility zones.

On the other hand, for the slope-based absence-data sampling, the Kaptai lake, its nearby areas, and some small patches in the southeast were classified as very low or low susceptibility zones. The visual comparison of the landslide susceptibility maps generated by the slope and MD-based methods shows that comparatively, more areas were classified as high or very high susceptibility zones for the slope-based sampling method than the MD-based sampling method. Some areas in the southeast of the area were classified as low or moderate susceptibility zones for the MD-based sampling method. Still, the same areas were classified as high or very high susceptibility zones in the slope-based sampling method. The areas close to the fault lines were classified as high or very high susceptibility zones in the slope-based sampling method, but only some patches in these areas were classified as very high and high susceptibility zones in the MD-based sampling method.

### Performance of landslide susceptibility maps

#### The ROC curve

**Figure 10:**
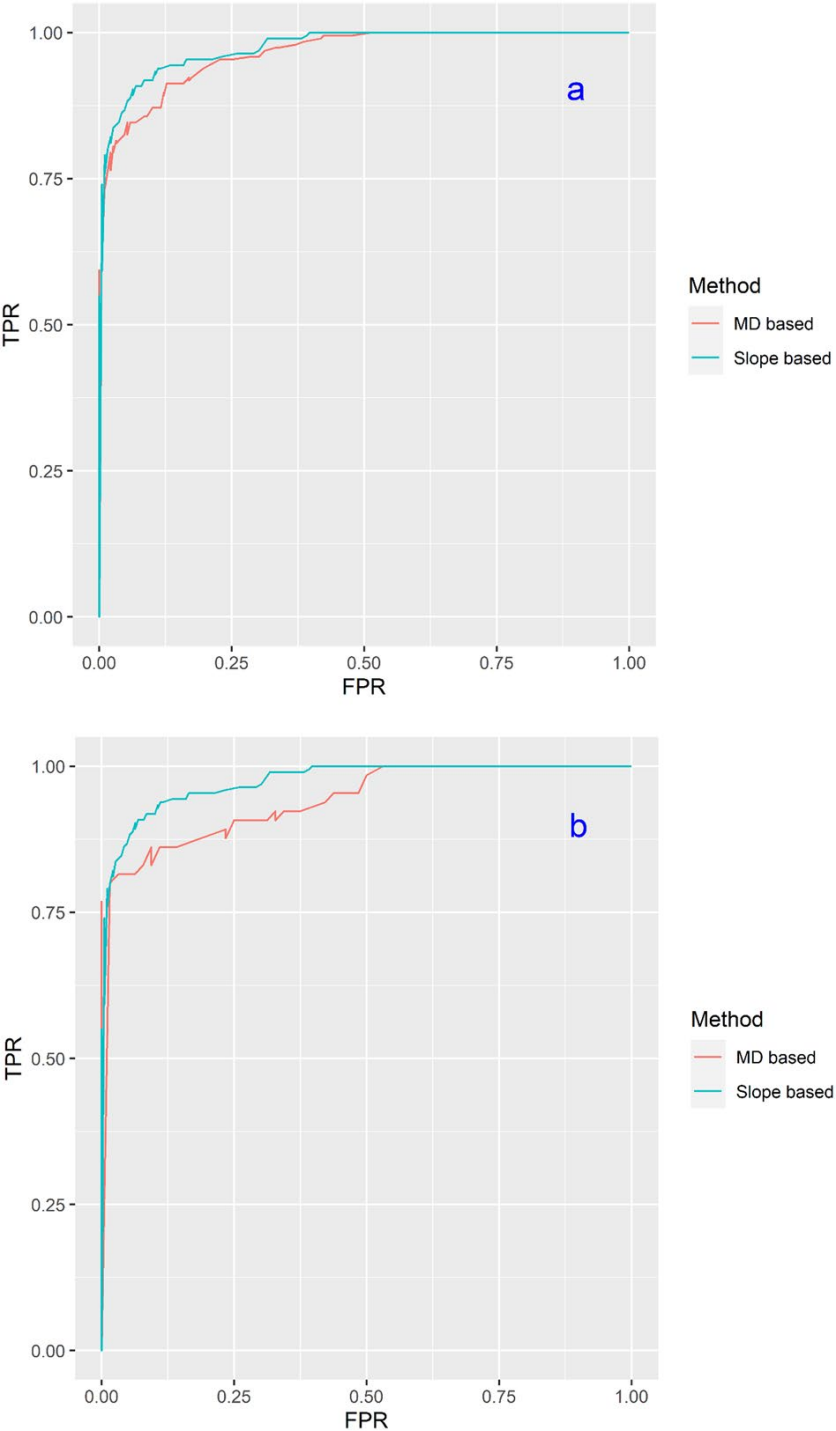
ROC curves for MD and slope based susceptibility maps: (**a**) training dataset (Slope based, AUC = 0.89; MD based AUC = 0.87); (**b**) validation dataset (Slope based AUC = 0.86; MD based AUC = 0.85).

The performance of MD-based and slope-based landslide susceptibility maps using the ROC curve is shown in Fig. 10. The AUCs for training dataset (Fig. 10a) for MD and slope-based sampling were 0.87 and 0.89, respectively. The AUCs for validation datasets (Fig. 10b) for MD and slope-based sampling were 0.85 and 0.86, respectively. It seems that the slope-based sampling method slightly outperforms the MD-based sampling. Nonetheless, the AUCs for both sampling methods are similar and fall in the good category of 0.8–0.9^44^. The visual comparison indicates that the map of the slope-based sampling method classified slightly more areas as high or very high susceptibility zones. However, it failed to differentiate low susceptibility from high susceptibility zones and classified most of the areas as high susceptibility zones, overestimating landslide susceptibility.

#### Statistical index based measures

The TPR and TNR values of the map produced by the MD-based sampling (Table 2) are 0.93 and 0.90, respectively, for the training data, indicating that this map has similar accuracy in differentiating the absence and presence data of landslides. These two values reduce to 0.88 and 0.89, respectively for the validation dataset, indicating similar performance in distinguishing absence and presence landslides for the unknown dataset. The Kappa values is > 0.8 for the training dataset, representing a strong agreement, it reduces to 0.77 for the validation dataset, representing a moderate agreement.

**Table 2:** Statistical measures of random forest model for different thresholds of Mahalanobis distance.

Sampling method	Dataset	TPR	TNR	Kappa
MD-based	Training	0.93	0.90	0.84
	Validation	0.88	0.89	0.77
Slope-based	Training	0.97	0.82	0.79
	Validation	0.96	0.82	0.78

In slope-based sampling for the training dataset, TPR and TNR (Table 2) were 0.97 and 0.82, respectively. Unlike the MD-based sampling method, slope-based sampling method showed better performance in detecting the landslides than the non-landslide locations. This model classified some non-landslide locations as landslides or gave false alarms. Kappa indices for the training validation dataset were 0.79 and 0.78, respectively. The AUC values for slope-based model were better than the MD-based model. But the Kappa value was better for MD-based model. It occurred because slope-based sampling had comparatively lower TNR than the MD-based sampling. MD-based model was efficient in detecting both presence and absence data whereas slope-based sampling showed low performance in detecting absence data. TPR was comparatively higher for slope—based model than the MD-based model.

#### Map consistency

SCAI assesses the consistency of the landslide susceptibility model. A high consistent model would have low SCAI values with the least percentage of the area classified as high susceptibility zones, but most of the existing landslides fall within these zones.

For the map generated using the MD-based sampling, around 58.0% of the study area were classified as very low and low susceptibility zones and approximately 35.0% of the study area were classified as high and very high susceptibility zones that contain around 78.0% of the existing landslides. The SCAI values decreased from 28.21 to 0.13 from very low to very high susceptibility zones. These results indicate that the susceptibility map is consistent and classified a significant portion of the area as very low and low susceptibility zones. The SCAI values are 0.13 for high susceptibility zones, indicating the model classified very few percentages of the area as very high susceptibility zones.

around 42.0% (Table 3) of the study area was classified as low or very low susceptibility zone in slope based. In contrast, around 46.0% of the study area was classified as either high or very high susceptibility zones. Compared to MD-based sampling, slope-based sampling classified almost two times more areas as high and very high susceptibility zones. Both slope and MD-based sampling gave similar accuracy. Still, landslide susceptibility based on a slope-based sampling classified almost half of the area as high and very high susceptibility zones. It indicates an overestimation of landslide susceptibility by the model. With the change of susceptibility, the SCAI value decreased. In the very high susceptibility zone, the SCAI value was 0.43, which is three times the SCAI value at that susceptibility zone in MD-based sampling. Therefore, the landslide susceptibility map produced using slope-based sapling is not as consistent and desirable as the MD-based sampling of absence-data.

**Table 3:** SCAI values for each susceptibility zones of Mahalanobis distance-based landslide susceptibility mapping.

Sampling method	Susceptibility	Area (%)	Landslide (%)	SCAI Index
Mahalanobis distance-based	Very low	33.57	1.19	28.21
	Low	24.87	4.76	5.22
	Moderate	19.34	15.87	1.22
	High	15.10	21.83	0.69
	Very High	7.12	56.35	0.13
Slope-based	Very Low	32.55	0.0	-
	Low	9.41	2.38	3.95
	Moderate	8.63	3.97	2.17
	High	15.67	13.10	1.20
	Very High	33.75	80.56	0.42

## Discussion

We proposed an objective MD-based absence-data sampling method and compared it with the slope-based method for landslide susceptibility mapping. The MD values were assumed to follow the Chi-square distribution. The threshold for absence-data sampling was then determined by the degree of freedom of the Chi-square distribution and a specific confidence level. Our results indicate that the absence sampling space spreads over the entire study area for our proposed method, avoiding the sampling bias towards any specific landslide locations. Although other distance-based matrices, like similarity index, have been used^21^, the critical value has been determined subjectively for the absence-data sampling. Our proposed method provides an objective and statistically robust means to determine the critical value based on the Chi-square distribution of the MD values of the landslide locations and a user-specified confidence level.

Slope-based sampling is commonly used in landslide susceptibility mapping^12,19,48^. Even though the slope is being used in determining the safe zone for absence data sampling it is used as a factor in the model. Slope plays the most crucial role in determining the landslide susceptibility of an area. However, unlike MD-based sampling, it is impossible to determine the critical value for the slope-based sampling based on our proposed method because the degree of freedom is zero. In our comparison study, the size of the sampling space based on the threshold of slope < 3° was comparatively larger than the MD-based sampling, but the sampling space was more clustered in the Kaptia lake and its nearby area. Therefore, the absence data were sampled only from these clustered areas. The slope-based sampling classified most areas as either very high or very low susceptibility zones. It also classified some landslide-free zones as vulnerable zones, overestimating the landslide susceptibility^8^. In addition, we notice that some studies have also included slope in the model, although it has already been used for absence-data sampling^12,13^ The use of slope in both absence-data sampling and landslide susceptibility modeling likely produces a biased model to slope. We recommend excluding the slope in landslide susceptibility model if it is used for absence-data sampling.

The ROC curves and statistical measures have been widely used for accuracy assessment, while the consistency and desirability of the map are commonly ignored^12,21,25,31^. Both accuracy and consistency should be assessed for landslide susceptibility mapping because a map may lose its consistency by continuously increasing the classified areas of high and very high susceptibility zones in order to achieve a high accuracy^26^. Our study showed that our proposed MD-based sampling method produces the landslide susceptibility map with satisfactory accuracy and consistency. In contrast, the slope-based sampling may damage the consistency by classifying most areas as high susceptibility zones^12,25^.

As mentioned, random sampling is the most common method for absence data sampling^20^. But in that case, there is a high chance that absence data will be sampled from an area which is highly prone to landslides or areas where landslides previously occurred. Moreover, it requires a very detailed landslide inventory and in some areas like the developing world a detailed inventory is not available. For such an area our proposed method will be helpful since prior to run the statistical or machine learning model based on MD we determine an area safe for absence data sampling.

Our proposed method reduces the subjectivity in choosing the threshold by comparing the MD values with the Chi-square distribution and applying a widely used statistical confidence level. In contrast, the determination of the slope threshold is subjective. Therefore, our proposed method is more statistically robust and scientifically viable than the slope-based sampling.

## Conclusions

This study proposed an objective MD-based absence-data sampling method for landslide susceptibility mapping. We compared our proposed method with a commonly used slope-based absence-data sampling in producing landslide susceptibility maps based on a random forest model. Our results indicate that the landslide susceptibility map produced using the MD-based method is satisfactory in accuracy and consistency. Our proposed approach is less subjective because the critical value was determined based on a Chi-square distribution and a user-specified significance level. On the other hand, the slope-based sampling is subjective and results in a biased model towards the slope. We recommend excluding the slope from the model if it is used in absence-data sampling. Although the slope-based method produces almost similar accuracy for landslide susceptibility map in terms of AUC, but the SCAI values indicated this method overestimates landslide susceptibility. Moreover, Kappa values also showed that MD-based absence data sampling provides better performance. The slope-based absence-data sampling method depends on the researcher's judgment and is based on one landslide causal factor. In contrast, multiple factors are used in MD-based absence-data sampling to determine the sampling space. Therefore, our proposed MD-based sampling method is more objective and statistically robust than the slope-based method. It can be used for landslide susceptibility mapping in other areas, especially where landslide inventory is not representative for the whole region.

## Data Availability

By request to authors and code for Mahalanobis distance method is available at https://github.com/yrabby/Mahalanobis-Distance-for-Raster-Files. https://doi.org/10.3390/data5010004.
